# Effects of seasonal precipitation change on soil respiration processes in a seasonally dry tropical forest

**DOI:** 10.1002/ece3.5912

**Published:** 2019-12-10

**Authors:** Shiqin Yu, Qifeng Mo, Yuanqi Chen, Yingwen Li, Yongxing Li, Bi Zou, Hanping Xia, Wang Jun, Zhian Li, Faming Wang

**Affiliations:** ^1^ Xiaoliang Research Station for Tropical Coastal Ecosystems Key Laboratory of Vegetation Restoration and Management of Degraded Ecosystems Guangzhou China; ^2^ The CAS engineering Laboratory for Ecological Restoration of Island and Coastal Ecosystems South China Botanical Garden Chinese Academy of Sciences Guangzhou China; ^3^ University of Chinese Academy of Sciences Beijing China; ^4^ Southern Marine Science and Engineering Guangdong Laboratory (Guangzhou) Guangzhou China; ^5^ College of Forestry and Architecture South China Agricultural University Guangzhou China; ^6^ Hunan Province Key Laboratory of Coal Resources Clean‐utilization and Mine Environment Protection Hunan University of Science and Technology Xiangtan China

**Keywords:** climate change, precipitation regime, rainfall change, soil CO_2_, tropics

## Abstract

Precipitation is projected to change intensity and seasonal regime under current global projections. However, little is known about how seasonal precipitation changes will affect soil respiration, especially in seasonally dry tropical forests. In a seasonally dry tropical forest in South China, we conducted a precipitation manipulation experiment to simulate a delayed wet season (DW) and a wetter wet season (WW) over a three‐year period. In DW, we reduced 60% throughfall in April and May to delay the onset of the wet season and irrigated the same amount water into the plots in October and November to extend the end of the wet season. In WW, we irrigated 25% annual precipitation into plots in July and August. A control treatment (CT) receiving ambient precipitation was also established. Compared with CT, DW significantly increased soil moisture by 54% during October to November, and by 30% during December to April. The treatment of WW did not significantly affect monthly measured soil moisture. In 2015, DW significantly increased leaf area index and soil microbial biomass but decreased fine root biomass. In contrast, WW significantly decreased fine root biomass and forest floor litter stocks. Soil respiration was not affected by DW, which could be attributed to the increased microbial biomass offsetting the decrease in fine root biomass. In contrast, WW significantly increased soil respiration from 3.40 to 3.90 μmol m^−2^ s^−1^ in the third year, mainly due to the increased litter decomposition and soil pH (from 4.48 to 4.68). The present study suggests that both a delayed wet season and a wetter wet season will have significant impacts on soil respiration‐associated ecosystem components. However, the ecosystem components can respond in different directions to the same change in precipitation, which ultimately affected soil respiration.

## Graphical abstract

The decoupling between soil respiration and soil moisture under seasonal precipitation change in a tropical forest highlights the complexity of how seasonal precipitation changes affect soil respiration.

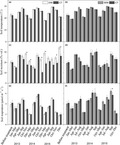

## 1 INTRODUCTION

Soil respiration is a vital flux that influences carbon (C) exchange between the soil and atmosphere (Bond‐Lamberty & Thomson, 2010; Schlesinger & Andrews, 2000), and as such changes in this flux could substantially affect the concentration of CO_2_ in the atmosphere (Bond‐Lamberty, Bailey, Chen, Gough, & Vargas, 2018). Rising atmospheric CO_2_ concentration leads to global warming, which in turn intensifies hydrologic cycles (Huntington, 2006) and further affecting changing in global and regional precipitation patterns (Min, Zhang, Zwiers, & Hegerl, 2011; Seneviratne et al., 2010). Soil moisture is among the most important factors regulating soil respiration (Gabriel & Kellman, 2014; Liu, Zhang, & Wan, 2009; Vicca et al., 2014).

Generally, soil respiration increases from low to medium soil moisture, reaches a plateau at optimum moisture, and declines at high soil moisture (Moyano, Manzoni, & Chenu, 2013; Xu, Baldocchi, & Tang, 2004). Previous studies suggest that soil respiration increases following increased precipitation or prolonged wet season, and decreases with decreased precipitation or drought (Liu, Wang, et al., 2016; Meir et al., 2015; Wu, Dijkstra, Koch, Penuelas, & Hungate, 2011). Excess soil water could induce O_2_ deficit and impede the diffusion of surface CO_2_ emission, which would finally depress the soil respiration (Kreuzwieser & Gessler, 2010; Makiranta, Minkkinen, Hytonen, & Laine, 2008; Moyano et al., 2013). Thus, soil respiration does not always respond positively to precipitation change. In some studies, drought has been found to increase soil respiration by alleviating the oxygen deficit (Liu, Liu, et al., 2016; Zhang et al., 2015). In contrast, increased precipitation has been found to decrease soil respiration in a mesic ecosystem (Suseela & Dukes, 2013). Furthermore, there are some studies suggesting that soil respiration is resistant to precipitation changes (e.g., Davidson, Nepstad, Ishida, & Brando, 2008; Deng et al., 2012; Zhang et al., 2015). For example, in a seasonally dry forest, Jiang et al. (2013) found that doubling precipitation did not affect soil respiration during the wet season when the ambient precipitation was high. Taken together, these studies suggest that though soil moisture can strongly regulate soil respiration, the direction and sensitivity of soil respiration response to precipitation change may still depend on both the water status of the studied ecosystem.

Precipitation can strongly affect soil moisture. However, soil texture, vegetation structure, and ambient climate can substantially mediate the effect of a precipitation on soil moisture after the precipitation event (Porporato, Daly, & Rodriguez‐Iturbe, 2004; Weil & Brady, 2017). As a result, soil moisture does not always respond proportionally to change in precipitation. For example, in a tropical rain forest, it was found that neither 25% nor 50% reduction in annual precipitation could significantly affect soil moisture (Cleveland, Wieder, Reed, & Townsend, 2010). In an arid grassland, 30% increase of annual precipitation did not change soil moisture (Zhao, Huang, Ma, Li, & Zhou, 2012). In a seasonally tropical forest, Zhou et al. (2011) found that even when annual total precipitation kept constant, seasonal changes in precipitation could lead to dramatic changes in soil moisture. Given that soil moisture is a principle way precipitation change affecting an ecosystem, the disproportionate response of soil moisture may introduce many uncertains on the estimation of precipitation change effects.

According to the source of C, soil respiration can be divided into several components (i.e., root respiration, soil microbial respiration, litter respiration), which are essentially driven by the metabolism of plants and soil decomposers (Hanson, Edwards, Garten, & Andrews, 2000; Kuzyakov, 2006; Kuzyakov & Larionova, 2005). Soil respiration is generally well correlated with root biomass (e.g., Huang et al., 2018; Wang, Yang, & Zhang, 2006), soil microbial biomass (e.g., Lee & Jose, 2003; Zhao, Wang, Cao, Zhao, & Gadow, 2018), and litter decomposition rates (e.g., Bowden, Nadelhoffer, Boone, Melillo, & Garrison, 1993; Wang, Yu, He, & Wang, 2017). In a subtropical forest, Jiang et al. (2013) showed that the variation of soil respiration responses to double annual precipitation between dry and wet seasons was coupled with the changes of fine root biomass and soil microbial biomass. In humid tropical forests, many studies attribute increased soil respiration after precipitation reduction to the enhanced soil microbial activity (Cleveland et al., 2010; Waring & Hawkes, 2015; Wood, Detto, & Silver, 2013). In drier ecosystems, reduction in precipitation is often found to inhibit the activity of decomposers and roots, and thus soil respiration (Sotta, Veldkamp, Schwendenmann, Guimarães, et al., 2007; van Straaten, Veldkamp, & Corre, 2011). On the other hand, soil and plant characteristics, such as soil texture (Meir et al., 2015) and plant community (Whitaker et al., 2014), could indirectly regulate soil respiration responses by altering the sensitivity of soil respiration to precipitation changes. As a result, the measurement of soil and plant characteristics in the studied ecosystem could contribute to the interpretation of the soil respiration response under precipitation changes.

Seasonally dry tropical forests are characterized by distinct wet and dry seasons, with 4–6 dry months (rainfall <100 cm) (Dirzo, Young, Mooney, & Ceballos, 2011), and comprise approximately 40% of the tropical forest lands (Murphy & Lugo, 1986). In East Asia, seasonally dry tropical forests are projected to receive more precipitation in the wet season and will have a one‐ or two‐month delayed wet season (Luo et al., 2008; Meehl, Arblaster, & Tebaldi, 2005; Zhou et al., 2011). As seasonal precipitation regimes are key factors regulating these forests’ ecosystem processes and functions (Jaramillo, MartÍnez‐YrÍzar, & Sanford, 2011; Parsons, Congdon, Storlie, Shoo, & Williams, 2012; Tunlid, Hoitink, Low, & White, 1989), substantial alteration in ecosystem processes may occur due to seasonal precipitation changes. Dry season irrigation in dry tropical forests has increased various biogeochemical processes, such as litter decomposition (Vasconcelos, Zarin, da Rosa, de Assis Oliveira, & de Carvalho, 2007; Wieder & Wright, 1995) and aboveground productivity (Vasconcelos, Zarin, Araujo, & I. d. S. Miranda., 2012; Wilson, Marra, & Sillett, 2013). However, there are few studies directly exploring the effects of seasonal precipitation changes on soil respiration (Allen et al., 2017). In this study, we delayed the wet season (DW) for two months by reducing throughfall during early wet season and then adding water during early dry season. To simulate a wet season receiving more precipitation, we established a wetter wet season treatment (WW, 30% increase of annual precipitation) by adding water during mid‐wet season. Our main objectives in this paper include (a) exploring the effect of DW and WW on soil respiration; (b) revealing how DW and WW affect soil respiration according to the responses of soil moisture and other soil respiration‐related plant and soil characteristics. We hypothesized that (a) in DW, the throughfall reduction in the early wet season would decrease soil moisture, negatively affect soil respiration and associated ecosystem components (such as microbial biomass and fine root biomass), while the water addition in the early dry season would increase soil moisture, positively affect soil respiration and the associated ecosystem components; (b) in WW, the water addition in the mid‐wet season would increase soil moisture, negatively affect the ecosystem components and depress soil respiration.

## 2 MATERIALS AND METHODS

### 2.1 Study sites

The precipitation manipulation experiment was conducted at the Xiaoliang Tropical Coastal Ecosystem Research Station (110°54′E, 21°27′N), Chinese Academy of Sciences, Guangdong Province, China. The climate here is a tropical climate with distinct wet (from April to September) and dry (from October to March) seasons. The mean annual temperature is 23°C. The mean annual precipitation is 1,400–1,700 mm, with more than 70% annual precipitation in the wet season. The soil is lateritic and developed from deeply weathered granite. Our experiment site was located in a seasonally dry tropical forest. The soil depth is over 1 m; the soil C content (0–100 cm) is less than 2%; the soil (0–60 cm) is sandy soil with 75.3% sand and 24.7% silt and clay. According to a vegetation investigation in 2015, the dominant tree species were *Aphanamixis polystachya*, *Schefflera octophylla*, *Carallia brachiate*, *Symplocos chunii*, *Acacia auriculaeformis*, *Photinia benthamiana*, and *Cinnamomum burmanni*, the dominant shrub and herb were *Dicranopteris dichotoma*, *Lygodium japonicum*, *Blechnum orientale, Psychotria rubra*, *Uvaria microcarpa*, and *Clerodendrum cyrtophyllum*.

### 2.2 Experiment design

In 2012, we established four experimental blocks in the tropical forest. Each experimental block consisted of a delayed wet season (DW), a wetter wet season (WW), and a control plot (CT). Each plot was 12 × 12 m^2^ and at least 3 m away from each other. In DW and WW, polyvinyl chloride (PVC) plates were inserted into the depth of 0.5 m along each plot's borders to prevent surface runoff and lateral movement of water from/into the surrounding soil. Transparent PVC shelters supported by stainless steel framework were constructed in DW plots. The transparent shelters were set at 2.5 m height. Understory sprinkling irrigation systems were constructed in both DW and WW plots. In each plot, the understory sprinkling irrigation systems consisted of 9 sprayers distributed uniformly. These sprayers were 1 m in height and connected to tap water pipelines. The water used for irrigation was groundwater from a nearby deep well.

From April to May, all the shelters were spread, which in total covered about 60% of the ground area in each DW plot. As a result, about 60% of throughfall would be intercepted by the shelters and runoff from DW plots. By doing this, the onset of wet season was expected to be delayed for two months. From October to November, each DW plot was irrigated once a week. In total, eight times of equivalent irrigation were conducted and the total amount of water added into the DW plots equaled the 60% of throughfall in the CT. This water addition manipulation was conducted to delay the end of wet season. The throughfall amount was determined using four cross‐shape throughfall traps located around the experimental sites in the same forest. A throughfall trap was 1.25 m^2^ in area, which was connected to a water meter for volumetric measurement. The throughfall amount excluded by the PVC shelters was determined by multiplying the total throughfall during April and May with 60%. Specifically, the amount of water added into a DW plot in the early dry season was about 245 mm, 90 mm, and 239 mm in 2013, 2014, and 2015, respectively. In WW plots, each plot was irrigated with 50 mm of water over a four‐hour periods every week during July and August; this occurred a total of eight times to simulate an approximate 25% increase in mean annual precipitation (1,600 mm). Precipitation manipulations in DW and WW were first carried out in 2013, and the same manipulation continued in 2014 and 2015. During the experimental period, CT plots received ambient precipitation inputs.

### 2.3 Soil respiration and soil moisture measurements

In each plot, three subplots were randomly located in the summer of 2012. A PVC collar (20 cm internal diameter, 5 cm height) was positioned and inserted 2 cm into the soil in each subplot. Each collar was placed at least 2 m away from the edge of plot to avoid edge trenching effect. The PVC collars were left in the same locations throughout the experiment. Soil respiration was measured using a soil CO_2_ flux system (Li‐8100, LI‐COR Ltd.) with a LI‐8100 chamber (model: 8100–103, LI‐COR Ltd.). Soil respiration was measured monthly to obtain mean soil CO_2_ flux (Suseela, Conant, Wallenstein, & Dukes, 2012; Wang et al., 2012). Before the precipitation manipulations started, soil respiration was measured in September and December in 2012. From January 2013 to December 2015, we took soil respiration measurement about once a month. Soil respiration measurements were carried out during 9:00–12:00 a.m. Every single measurement in one subplot (one collar) took 2 min. To avoid the interruption from pulse soil respiration induced by rainfall, we took measurement at least 48 hr after rainfall events (if there was any). The aboveground parts of living plants inside the collars (if there was any) were removed by hand, to carefully eliminate aboveground plant photosynthesis and respiration. Litter fallen into the collars was left in the collars to include CO_2_ released from litter decomposition. In a typical monthly measurement, there are 12 measurements for a treatment. The values of three subplots were averaged as one replicate.

Soil temperature (0–10 cm) was measured using an Omega soil temperature probe (model: 8100–201, LI‐COR Ltd.). Soil moisture (volumetric water content) was measured with a soil moisture probe (model: 8100–204, LI‐COR Ltd.). Both soil temperature and soil moisture were measured outside the collars when soil respiration was measured. The values of three subplots were averaged as one replicate.

### 2.4 Plant and soil characteristic measurements

Most plant and soil characteristic measurements were conducted in January, April, August, and November of 2015, representing the late dry season, early wet season (when throughfall was reduced to delay the onset of the wet season in DW), mid‐wet season (when water was added in WW), and early wet season (when water was added to extend the end of the wet season in DW), respectively. Similarly, to reduce trenching effects and interferences from nearby plots, all soil and plant samples were collected at least 2 m away from the edge of plot. Leaf area index was measured using a Plant Canopy Analyzer (LAI 2000, LI‐COR Ltd.). At the beginning of measurements of a plot, ten measurements were taken above the canopy level in a nearby open field, and then, at least 30 measurements were taken below the canopy level in a plot. Leaf area index was measured before sunrise on days without rainfall. The standing understory biomass was measured through harvesting. In January 2015, two 1 × 1 m^2^ subplots were randomly selected and the aboveground standing biomass in the subplots was harvested. In the later harvests, to reduce disturbances, we only harvested aboveground vegetation within two 0.5 × 0.5 m^2^ subplots in each plot. The harvested vegetation was dried at 60°C in laboratory until reaching a constant weight.

Typically, CO_2_ derived from microbes and plant roots in surface soil makes up a majority of total soil respiration (Luo & Zhou, 2006). As a result, surface soil and root samples (0–10 cm) were taken and analyzed to represent the soil and plant root responses. In each soil sampling, six cores of soil samples were collected using a drill sampler (5 cm internal diameter) in each plot. Live fine root (diameter ≤2 mm) was picked out, washed to remove the soil attached on surface, and dried at 60°C for 48 hr and weighed. The weight was considered fine root biomass. After removing roots and other plant residues, the soil samples were immediately sieved through a 2‐mm mesh sieve. Soil dissolved organic C was extracted in K_2_SO_4_ solution and analyzed using a TOC analyzer (TOC‐VCSH, Shimadzu GmbH). Soil microbial biomass was estimated by measuring microbial biomass C using the fumigation‐extraction method (Vance, Brookes, & Jenkinson, 1987). Specifically, dissolved soil organic C was extracted using 0.5 M K_2_SO_4_ before and after 48 hr of chloroform fumigation. Carbon concentrations in extracts were determined on the Shimadzu TOC analyzer. The difference of C concentrations was used for calculating the microbial biomass C. Soil pH was determined in 1:2.5 (g:mL) mixture of soil: deionized water.

The standing litter mass on forest floor was determined in January and November of 2015. In each sampling, two 1 × 1 m^2^ subplots were randomly selected in a plot. All aboveground leaf litter and woody debris (diameter <2 cm) were collected and dried at 60°C in laboratory until the litter reached a constant weight. Soil organic C was measured in January of 2015 using the H_2_SO_4_‐K_2_Cr_2_O_7_ oxidation method (Liu, Jiang, Zhang, & Liu, 1996).

### 2.5 Data analysis

The normality was tested with the method of Shapiro–Wilk. We carried out a mixed‐model analysis using the restricted maximum likelihood (REML) estimation procedure to identify any main and interactive effects of treatments and periods (month/year) on soil temperature, soil moisture, and soil respiration in each experimental period, with each monthly measurement as a repeated measurement (Zuur, Ieno, & Smith, 2007). The mixed‐model analysis was also used to examine the effect of treatments and periods on plant and soil characteristics including leaf area index, understory biomass, fine root biomass, microbial biomass C, dissolved organic C, pH, and forest floor litter stock, with each sampling as a repeated measurement. In addition, we used a mixed‐model analysis to examine the treatment effect on soil respiration in each year with each monthly measurement as a repeated measurement. The relationship between soil respiration change due to DW/WW and time was examined using a linear regression. The temperature sensitivity and moisture sensitivity of soil respiration were examined and followed by Suseela et al. (2012). The fitness of all the regression models was reviewed by both quantitative and graphic methods. We conducted one‐way ANOVA to examine any differences in plant and soil characteristics among treatments in each sampling regime. Least significant difference (LSD) tests were used to detect any differences among individual treatments. All data analyses were performed in SPSS 20.0 (IBM Corp).

## 3 RESULTS

### 3.1 Monthly precipitation from 2012 to 2015

**Figure 1:**
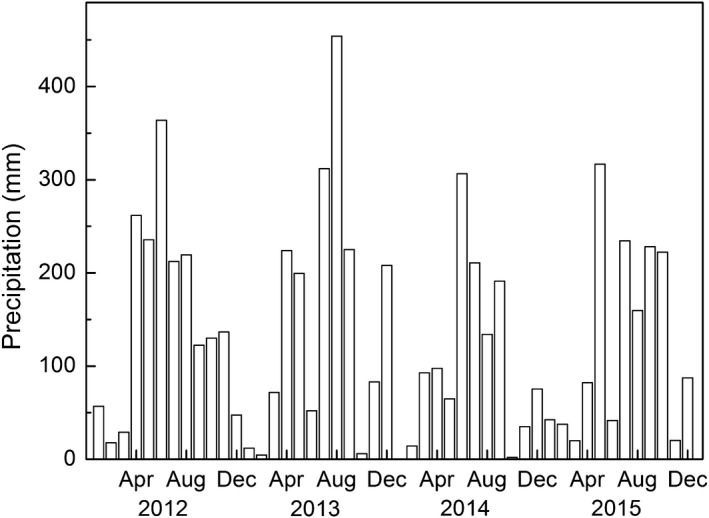
Monthly precipitation from 2012 to 2015 at Xiaoliang Research Station, Guangdong, PR, China

Annual precipitation was 1,833.2, 1,852.2, 1,224.7, and 1,493.3 mm from 2012 to 2015, respectively (Figure 1). Precipitation during wet season (April to September) accounted for 77.2%, 79.2%, 82.1%, and 71.2% of annual precipitation in 2012, 2013, 2014, and 2015, respectively. The highest monthly precipitation in each year was 363.9 mm, 454.0 mm, 306.4 mm, and 316.7 mm in June of 2012, August of 2013, June of 2014, and May of 2015, respectively. During dry season (October to March), monthly precipitation ranged from 2 to 208 mm. Most of the months received precipitation <100 mm. The highest monthly precipitation was observed in December of 2013.

### 3.2 Temporal dynamics of soil temperature, soil moisture, and soil respiration

Soil temperature typically peaked in June or July and reached a minimum in January or February (Figure S1a). Soil volumetric moisture ranged from 11.8% to 35.5%, 10.6 to 30.1%, and 10.1 to 30.4% in DW, WW, and CT, respectively, along the three‐year experiment (Figure S1b). In CT, the highest soil moisture in 2013, 2014, and 2015 was observed in June (29.0%), September (30.4%), and October (28.0%), while the lowest soil moisture was observed in January (14.0%), April (11.6%), and July (10.1%), respectively. In WW, the highest soil moisture in 2013, 2014, and 2015 was observed in May (30.1%), September (29.6%), and September (28.1%), while the lowest soil moisture was observed in March (12.9%), October (12.9%), and July (10.6%), respectively. In DW, soil moisture peaked in November 2013 (33.5%), September 2014 (32.9%), and December 2015 (35.5%), while the lowest soil moisture was observed in January (15.1%), April (11.8%), and July (14.4%) in 2013, 2014, and 2015, respectively. Soil respiration showed a clear seasonal pattern, which was in pace with the soil temperature dynamics (Figure S1c). Soil respiration was strongly correlated with soil temperature (*R* ^2^ = .567, *p* < .001) (Figure S2a). The temporal variation of soil respiration was also significantly explained by soil moisture, but with a low *R* ^2^ (*R* ^2^ = .030, *p* = .001) (Figure S2b).

**Figure 2:**
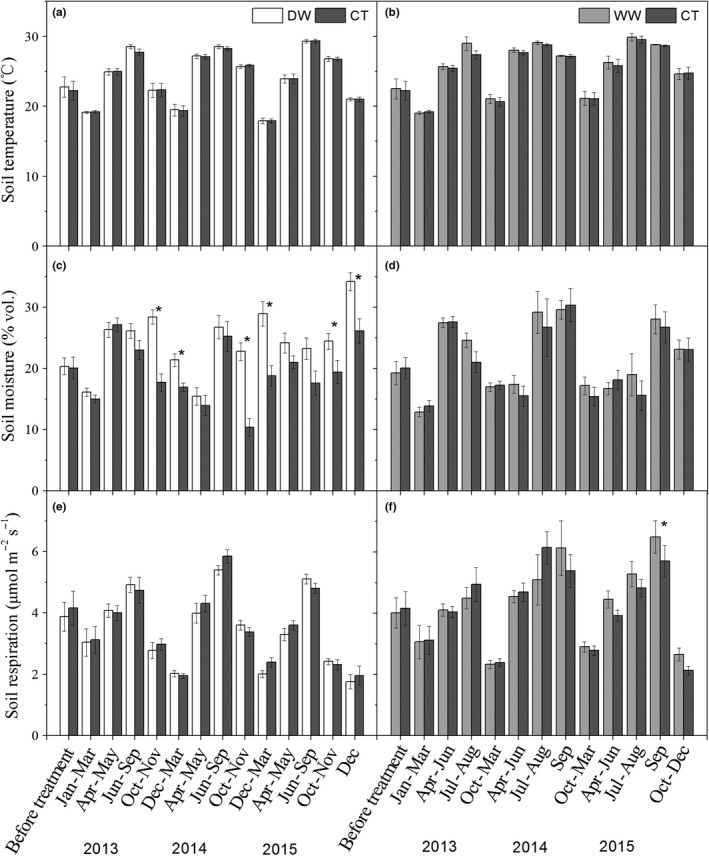
Mean soil temperature (a, b), soil moisture (c, d), and soil respiration (e, f) in DW, WW, and CT plots in each experimental period. * indicates that the difference between treatment and CT is significant at *α* = .050 level. The blocks of time were merged together depending on the climate attributes and precipitation manipulation

We did not find any significant difference in soil temperature among treatments in any period (Figure 2a,b). The difference in soil moisture between DW and CT before precipitation manipulation (2012) was not significant (*p* = .912, Figure 2c). Compared with the CT, 60% throughfall reduction in DW plots in the period of April to May did not significantly affect soil moisture in 2013 (*p* = .633), 2014 (*p* = .504), and 2015 (*p* = .123). Water addition in the period of October to November significantly increased soil moisture in those months and the subsequent months in dry season. In 2013, water addition increased soil moisture by 56.8% (*p* < .001) from October and November, and increased soil moisture by 20.4% (*p* < .001) from December to March. In 2014, water addition increased soil moisture by 107.7% (*p* < .001) from October to November. In 2015, water addition also increased soil moisture by 25.8% (*p* < .041) in the period of October and November and December by 30.8% (*p* < .017). In total, DW increased soil moisture in dry season. The difference in soil moisture between WW and CT before treatment was not significant (Figure 2d). In WW, water addition tended to increase soil moisture in July and August 2013 (*p* = .100) but had a minor effect on the soil moisture in other periods.

We did not detect any significant difference in soil respiration in any period between CT and DW (Figure 2e). In WW, we found that soil respiration was significantly higher than soil respiration in CT in September of 2015 (*p* = .029, Figure 2f). In addition, soil respiration of WW from October to December in 2015 tended to be higher than soil respiration in CT (*p* = .060).

### 3.3 Soil temperature, soil moisture, and soil respiration in each year

**Figure 3:**
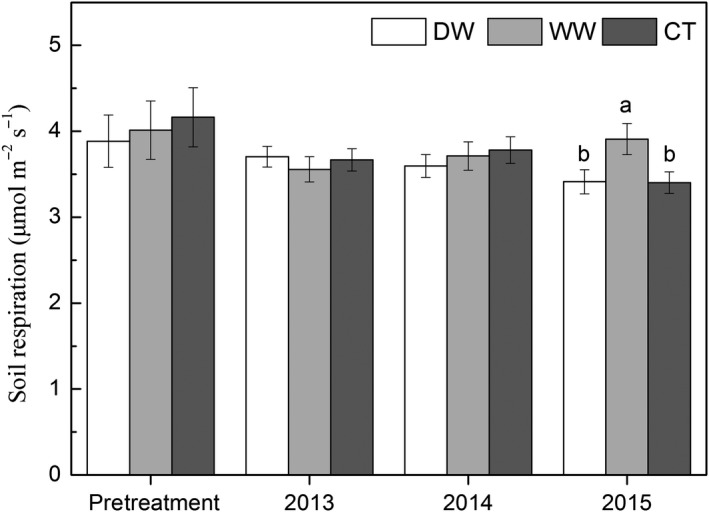
Mean soil respiration in each year in DW, WW, and CT. Different letters indicate significant differences at *α* = .050 level

There were not any significantly interactive effects of seasonal precipitation changes and year on either soil temperature (*p* = .994) or soil moisture (*p* = .934, Table 1). Precipitation changes did not significantly affect soil temperature (*p* = .965); however, they did significantly affect soil moisture (*p* < .001). The treatment effect on soil respiration significantly varied with year (*p* = .004). In 2015, the difference in soil respiration between CT and WW was significant (*p* = .021, Figure 3).

**Table 1:** Main and interactive effects of treatment and year on soil temperature, soil moisture, and soil respiration

	Soil temperature		Soil moisture		Soil respiration	
	*F*	*p*	*F*	*p*	*F*	*p*
Treatment	0.036	.965	11.881	<.001	0.518	.612
Year	5.518	.004	5.775	.004	4.121	.021
Treatment × Year	0.056	.994	0.208	.934	5.565	.004

### 3.4 Plant and soil characteristics

There was a significant interactive effect of precipitation changes and sampling month on leaf area index (Table 2). Compared to CT, DW significantly increased leaf area index by 40.1% in January (*p* = .034), but did not have significant influences in other months (Table 3). The precipitation changes significantly affected fine root biomass (*p* = .017), microbial biomass (*p* < .001), pH (*p* = .048), and forest floor litter mass (*p* = .046, Table 2). Compared to CT, DW decreased fine root biomass by 17.3% (*p* = .037) and increased microbial biomass C by 20.4% (*p* = .034, Table 3). The effect of DW on microbial biomass C in different periods was identical (*p* = .266), and the largest difference occurred in November (35.2%, *p* = .011). WW significantly increased pH (*p* = .002), but decreased fine root biomass (*p* < .001) and forest floor litter stock (*p* = .048). It was noted that fine root biomass in WW was 46.7% (*p* = .025) and 44.4% (*p* = .016) lower than that in CT in January and April, respectively.

**Table 2:** Main and interactive effects of treatment and month on plant and soil characteristics

	Treatment		Period		Treatment × Period	
	*F*	*p*	*F*	*p*	*F*	*p*
LAI	14.133	.002	55.697	<.001	15.448	.002
UB	1.013	.401	1.028	.333	0.092	.913
FRB	6.636	.017	0.231	.640	0.525	.609
MBC	22.592	<.001	26.413	<.001	1.509	.266
DOC	3.594	.071	140.742	<.001	2.542	.133
pH	4.352	.048	132.567	<.001	1.036	.393
SOM	3.448	.077	0.001	.981	0.378	.695
FL	1.732	.046	2.095	.176	0.468	.641

Note

Different letters indicate significant differences among treatments at *α* = .050 level.

Abbreviations: DOC, dissolved organic C (mg/kg dry soil); FL, forest floor litter mass (g/m^2^); FRB, fine root biomass (g/m^2^); LAI, leaf area index; MBC, microbial biomass C (mg/kg dry soil); SOM, soil organic matter content (%); UB, understory biomass (g/m^2^).

**Table 3:** Plant and soil characteristics in each period and the mean in DW, WW, and CT in 2015

	January	April	August	November	Average
LAI					
DW	3.68 (0.21)^a^	3.70 (0.12)	4.12 (0.05)	3.67 (0.10)	3.79 (0.13)^a^
WW	2.40 (0.16)^b^	2.90 (0.20)	4.10 (0.24)	3.46 (0.16)	3.22 (0.25)^b^
CT	2.61 (0.14)^b^	3.04 (0.17)	4.17 (0.10)	3.37 (0.11)	3.30 (0.21)^b^
UB					
DW	232.8 (18.6)	343.1 (24.0)	338.7 (19.8)	294.5 (17.0)	302.2 (22.3)^b^
WW	315.3 (19.1)	409.1 (30.0)	397.6 (27.6)	354.0 (16.8)	369.0 (24.2)^a^
CT	311.3 (36.5)	384.3 (20.4)	376.6 (34.3)	336.2 (33.7)	352.1 (29.9)^ab^
FRB					
DW	157.3 (8.35)^ab^	153.3 (10.5)^ab^	172.6 (9.3)	162.8 (15.7)	161.5 (10.4)^b^
WW	101.0 (13.4)^b^	114.0 (12.3)^b^	151.0 (12.9)	129.0 (9.8)	123.8 (12.2)^c^
CT	189.5 (17.2)^a^	204.8 (14.5)^a^	199.7 (21.4)	187.5 (15.1)	195.4 (15.6)^a^
MBC					
DW	428.7 (12.4)	555.2 (19.6)^a^	525.4 (9.6)^a^	349.5 (8.3)^a^	464.7 (27.0)^a^
WW	378.9 (9.6)	401.0 (21.9)^b^	387.1 (24.6)^b^	218.4 (10.6)^b^	346.4 (27.3)^b^
CT	373.7 (31.1)	457.1 (23.1)^ab^	455.0 (30.1)^ab^	258.4 (14.8)^b^	386.1 (33.3)^b^
DOC					
DW	311.6 (26.2)	395.2 (4.1)	312.5 (8.2)	265.2 (5.4)	321.1 (18.8)^a^
WW	232.3 (15.2)	356.6 (9.7)	266.8 (12.3)	203.2 (8.9)	264.7 (20.2)^b^
CT	239.2 (6.3)	357.8 (22.1)	311.8 (12.1)	260.3 (16.6)	292.3 (19.4)^ab^
pH					
DW	4.35 (0.04)^b^	4.31 (0.05)^b^	4.58 (0.03)	4.46 (0.03)^b^	4.42 (0.05)^b^
WW	4.58 (0.05)^a^	4.63 (0.07)^a^	4.79 (0.06)	4.71 (0.05)^a^	4.68 (0.06)^a^
CT	4.34 (0.02)^b^	4.40 (0.02)^ab^	4.54 (0.03)	4.63 (0.04)^ab^	4.48 (0.04)^b^
FL					
DW	869.1 (136.8)			1,096.5 (126.3)	982.8 (129.2)^a^
WW	628.3 (78.1)			853.7 (117.8)	741.0 (101.9)^b^
CT	929.9 (84.3)			943.9 (81.4)	936.9 (76.8)^a^
SOM					
DW	3.31 (0.09)				3.31 (0.08)
WW	3.25 (0.14)				3.25 (0.12)
CT	2.52 (0.33)				2.52 (0.29)

Note

Numbers in brackets are standard errors (*n* = 4). Different letters indicate significant differences among treatments at *α* = .050 level.

Abbreviations: DOC, dissolved organic C (mg/kg dry soil); FL, forest floor litter mass (g/m^2^); FRB, fine root biomass (g/m^2^); LAI, leaf area index; MBC, microbial biomass C (mg/kg dry soil); SOM, soil organic matter content (%); UB, understory biomass (g/m^2^).

The results of Pearson correlation suggested that soil moisture was significantly correlated with microbial biomass across treatment except in August (Table S1). However, most of the measured plant and soil characteristics were not correlated with soil moisture in any period.

## 4 DISCUSSION

### 4.1 Effects on soil moisture

As expected, water addition in early dry season significantly increases soil moisture by 25.8%–107.7%, and the effects lasted extensively into the subsequent months (Figure 2c). However, soil moisture did not change with either throughfall reduction in early wet season or water addition in mid‐wet season as expected (Figure 2c,d). Similar disproportional soil moisture responses to precipitation manipulation have been observed in several previous studies (Chou, Silver, Jackson, Thompson, & Allen‐Diaz, 2008; Cleveland et al., 2010; Zhao et al., 2017). High precipitation may drive soil to its maximum water holding capacity, which would eliminate the difference of soil moisture. In addition, difference in evapotranspiration due to different soil moistures can also eliminate difference of soil moisture. This process is slow and positively correlated with air and soil temperature. As a result, the cool (below 20°C) and dry climate in dry season in our tropical forest may influence the water addition in early dry season to last longer than expected. Consistent with our result, a three‐year experiment in a North American grassland found that increased precipitation in early growing season increased soil moisture for a whole‐growing season in the year of low annual precipitation (Chou et al., 2008). High ambient precipitation may drive soil up to their maximum water holding capacity even though throughfall was partially excluded. Cleveland et al. (2010) found that neither 25% nor 50% throughfall reductions influenced soil moisture in a one‐year experiment in tropical rainforest, which received about 3,870 mm/year throughfall, with monthly throughfall over 100 mm. In April and May of 2013, we recorded five rainfall events bringing more than 30 mm precipitation per day (data not shown), which could eliminate the throughfall reduction effect on soil moisture. In addition, the water addition in the previous dry season may significantly increase soil moisture during the entire dry season (Figure 2), which may also mask the effect of throughfall reduction in the early wet season. Due to the high precipitation in the wet season, soils are generally water saturated in July and August, and the added water in WW plots would leach out from this hilly forest by surface and lateral flow. It is thus reasonable that WW did not change soil moisture in this forest.

### 4.2 Effects on plant and soil characteristics

In line with many previous studies (Liu et al., 2009; Ren et al., 2018), our results showed that precipitation change increased soil microbial biomass by increasing soil moisture (Table S1). DW strongly increased LAI in January but barely affected it in August and November (Table 3). Precipitation can be an important factor influencing LAI. For example, in a Douglas fir forest increased precipitation (+100%) of growing season increased LAI by 12% during a two‐year experiment (Gower, Vogt, & Grier, 1992). In a moist tropical forests, about 75% throughfall reduction decreased LAI in the second year of the experiment (Nepstad et al., 2002). The LAI response pattern suggests that LAI was limited by soil moisture in the dry season. However, the response of LAI lagged behind soil moisture increasing. In an Amazon tropical forest, Vasconcelos et al. (2012) found higher dry season rainfalls resulted in higher ANPP increments during the following year, which also indicated a lag effect on plant productivity. Similarly, a lag effect of precipitation on stem growth was also reported in a dendrochronological study for a tropical tree species in Bolivia (Brienen & Zuidema, 2005). Fine root biomass decreased in DW (Table 3). It is well documented that plants tend to invest less biomass in fine roots with higher MAP (Belaytedla, Zhou, Su, Wan, & Luo, 2009; Markesteijn & Poorter, 2009), and moderate droughts have been found to induce more belowground C allocation (Leuschner et al., 2001; Liu et al., 2017; Steinberg, Miller, & McFarland, 1990). In a moist tropical forest, dry season irrigation decreased fine root biomass in 0–30 cm depth from 372 ± 63 g/m^2^ to 286 ± 39 g/m^2^ (Yavitt & Wright, 2001). In a dry forest in New Mexico, USA, double precipitation in the growing season also significantly decreased fine root biomass (Gower et al., 1992). In the present study, our LAI and fine root data suggested that increased soil moisture in DW alleviated water limitation of plants in the dry season, and thus, plants allocated less photosynthate to fine roots while investing more C to aboveground growth.

WW significantly reduced forest floor litter stock and fine root biomass but increased soil pH (Table 3). Precipitation change can affect ecosystems through dissolved organic matter (DOM) leaching (Deng et al., 2018; Inamdar, Christopher, & Mitchell, 2004; Maes & Steppe, 2012). A study in a tropical forest showed that high rainfalls enhanced readily decomposable soluble C that was transported from the forest floor to the soil (Wieder, Cleveland, & Townsend, 2009). Deng et al. (2018) also found that increased precipitation frequency enhanced litter DOM leaching in a tropical forest. The water addition in WW may increase litter DOM leaching and accelerate aboveground litter decomposition, which should contribute to the decreased forest floor litter mass. Reasons contributing to the decreased fine root biomass in WW may be different from those in DW, because water additions in WW were conducted in mid‐wet season when plants should not be limited by water availability. Though it was not observed, the water addition may induce short‐term water logging in July and August when the ambient soil moisture is high. The water logging may decrease fine root biomass, as O_2_ and CO_2_ diffusion are reduced (Leppälammi‐Kujansuu, Salemaa, Kleja, Linder, & Helmisaari, 2014; McCormack & Guo, 2014). In a subtropical evergreen forest, 70% rainfall reduction decreased soil pH in the first year of experiment (Bu et al., 2018). In a Mongolia steppe, water addition resulted in a significant increase in soil pH (Ma et al., 2016). These studies, combined with ours, suggest that soil pH can increase with increased precipitation.

### 4.3 Effects on soil respiration

In the present study, annual mean soil respiration was within the range between 1.68 and 4.14 μmol m^−2^ s^−1^ (1,286 ± 633 g C m^−2^ year^−1^) reported for tropics in a synthesis study (Bond‐Lamberty & Thomson, 2010). Soil temperature strongly accounted for the seasonal variation of soil respiration, whereas soil moisture also explained a part (Figure S2). The relationship between soil respiration and soil temperature/moisture also coincides with most of other studies (Gaumont‐Guay et al., 2006; Lloyd & Taylor, 1994; Yu et al., 2017).

Soil moisture is important for evaluating the effect of precipitation changes on soil respiration (Gabriel & Kellman, 2014; Liu et al., 2009; Wu et al., 2011). In the present study, DW significantly increased soil moisture, but did not affect soil respiration (Figure 2c,e). In contrast, WW did not change monthly measured soil moisture, but significantly increased soil respiration (Figure 2d,f). Several precipitation manipulation experiments also suggested that soil respiration was not affected by increased soil moisture (e.g., Davidson et al., 2008; Deng et al., 2012; Zhang et al., 2015). The decoupling between soil respiration and soil moisture change could be attributed to limitation of other environmental variables to soil respiration (Vasconcelos et al., 2004; Zhang et al., 2015), the adaption of soil microbes and plants to altered soil moisture (Bouskill et al., 2013; Kozlowski & Pallardy, 2002; Kruse, Turnbull, & Adams, 2012), and so on.

Soil respiration usually increases with fine root biomass (Jiang et al., 2013; Sotta, Veldkamp, Schwendenmann, Guimarães, et al., 2007; van Straaten et al., 2011), soil microbial biomass (Huang et al., 2018; Wang et al., 2006), and canopy photosynthesis (Doughty et al., 2015; Hogberg et al., 2001; Kuzyakov, 2010). The present study shows that DW increased microbial biomass and LAI but decreased fine root biomass (Table 3). Soil heterotrophic respiration could respond differently to precipitation changes with autotrophic respiration (Huang et al., 2018; Liu, Liu, et al., 2016; Lu et al., 2017). For example, Huang et al. (2018) found throughfall exclusion reduced soil heterotrophic respiration but increased autotrophic respiration in the dry season. In a moist tropical forest, Davidson, Ishida, and Nepstad (2004) found soil respiration did not change after throughfall exclusion, mainly due to the counteracting effects of fine root death and consequent enhanced microbial activity. These studies indicate that different soil respiration components/ecosystem components could respond to an identical precipitation change in opposite directions. In the present study, DW may increase soil microbial respiration through increasing microbial biomass, but decrease soil root respiration as fine root biomass decreased, which could explain the apparent unchanged soil respiration under the DW treatment.

DOM leaching into the soil is an important substrate source for soil organisms. In a tropical forest, Cleveland et al. (2010) found both 25% and 50% precipitation exclusion increased soil respiration via increasing litter DOM concentration. Similarly, Deng et al. (2018) found that increased precipitation frequency increased soil respiration by enhanced litter DOM leaching loss. As discussed above, WW could accelerate aboveground litter decomposition, which may result from the enhanced litter DOM leaching (Wieder et al., 2009). As a result, WW can increase soil respiration through transferring more litter DOM into the soil. WW also decreased fine root biomass (Table 3), which suggests a decreased plant root respiration. Moreover, the increased pH can also contribute to the increased soil respiration because soil respiration usually increases with pH when the pH is less than 7 as soil microbial activity increases (Kowalenko, Ivarson, & Cameron, 1978). The increased soil respiration indicates that the enhancement in litter DOM input and increased soil microbial activity had a stronger effect on soil respiration than the reduction in root biomass.

### 4.4 Limitation of this study

In the present study, we conducted monthly measurements of soil moisture and soil respiration and focused on the long‐term (months to years) effect of seasonal precipitation changes. It is possible that there were short‐term (hours to days) effects of the water treatments that were missed due to the low frequency of measurements. Although the soils are likely well drained due to the hilly land surface of this forest, water addition in the WW could have induced water logging in July and August when the ambient soil moisture was high. Short‐term water logging can depress soil CO_2_ and O_2_ diffusion thus directly decreasing soil respiration (Hall, McDowell, & Silver, 2013), which would recover as soon as water logged was drained off. In DW, throughfall exclusion in early wet season and water addition in early dry season may change the frequency and strength of pulse soil respiration induced by dry soil rewetting (“Birch effect”) (Griffiths & Birch, 1961; Lado‐Monserrat, Lull, Bautista, Lidon, & Herrera, 2014). Under the present experimental design of monthly measurements, such changes in both soil moisture and soil respiration cannot be estimated because these responses last for only hours to days. Thus, the dynamic nature of soil respiration and the net effect on annual soil respiration in these plots are likely not captured in the monthly measures.

In this study, we measured soil and plant characteristics in the surface soil. However, soil moisture, soil microbes, and plant roots in surface soil may respond differently to precipitation change from those in deep soil (Sotta, Veldkamp, Schwendenmann, Guimaraes, et al., 2007; Weil & Brady, 2017). As a result, the conclusion about the relationships between soil respiration and the environmental factors drawn from our results should be further studied in deeper soil profiles.

We did not insert PVC plates into the soil around CT plot as we did in other treatment plots, which introduced differences in the roots in surface soil and hydrology between CT and precipitation change plots. In addition, some plots within a block were only 3 m away from each other. There could be coarse roots that extend beyond 3 m in the soil below 0.5 m (the depth of trenching), which can obtain water from other treatment plots. To reduce such treatment weaknesses, we avoided making any samples and measurements near plot edges (distance >2 m). However, some uncertains might still remain in the comparison among treatments. In this study, the actual precipitation intercepted by precipitation roofs was not measured. It was not clear that whether the precipitation roofs were effective in precipitation interception, which could be a potential reason contributing to the unchanged soil moisture in the early wet season after the 60% throughfall exclusion.

## 5 CONCLUSIONS

Our field precipitation manipulation experiment showed that delaying the wet season for 2 months did not change monthly measured soil respiration even when soil moisture substantially increased. In contrast, 25% increase of mean annual precipitation in the mid‐wet season did not affect monthly measured soil moisture but increased soil respiration in the third year of the experiment. Both DW and WW significantly affected soil respiration‐associated ecosystem components. However, responses to the precipitation changes differed among different ecosystem components, which mediated the soil respiration responses. We found DW increased soil microbial biomass and LAI and decreased fine root biomass, while WW increased soil pH and decreased fine root biomass and forest floor litter stock. The responses of the ecosystem components indicate that (a) DW increased soil microbial respiration but decreased fine root respiration, which explains the apparent unchanged soil respiration and decoupling between soil moisture and soil respiration; (b) WW increased aboveground litter decomposition and soil microbial activity, which contributes to the increased soil respiration.

To gain a better understanding of how precipitation change regulates soil respiration, we suggest that future studies can explore the responses of different soil respiration‐associated ecosystem components such as fine root biomass, soil microbial biomass, litter decomposition rate, and soil pH. In addition, soil moisture and soil respiration should be monitored under a higher frequencies to integrate their pulsed and short‐term responses to the estimate on the effects of precipitation change.

## CONFLICT OF INTEREST

None declared.

## AUTHOR CONTRIBUTIONS

LZA and WFM designed the experiments. YSQ, MQF, LYW, LYX, ZB, and WJ performed the experiments. YSQ, LZA, and WFM analyzed the data. YSQ and WFM wrote the manuscript. LZA, WFM, CYQ, MQF, and XHP revised the manuscript.

## Data Availability

No data are associated with this manuscript.
