# Supplementary material for: Effects of seasonal precipitation change on soil respiration processes in a seasonally dry tropical forest
Source: Ecol Evol. 2019 Dec 10;10(1):467–79. doi: 10.1002/ece3.5912 (PMC6972815; doi:10.1002/ece3.5912)
Supplement: Supplementary file 1 [file ECE3-10-467-s001.docx]

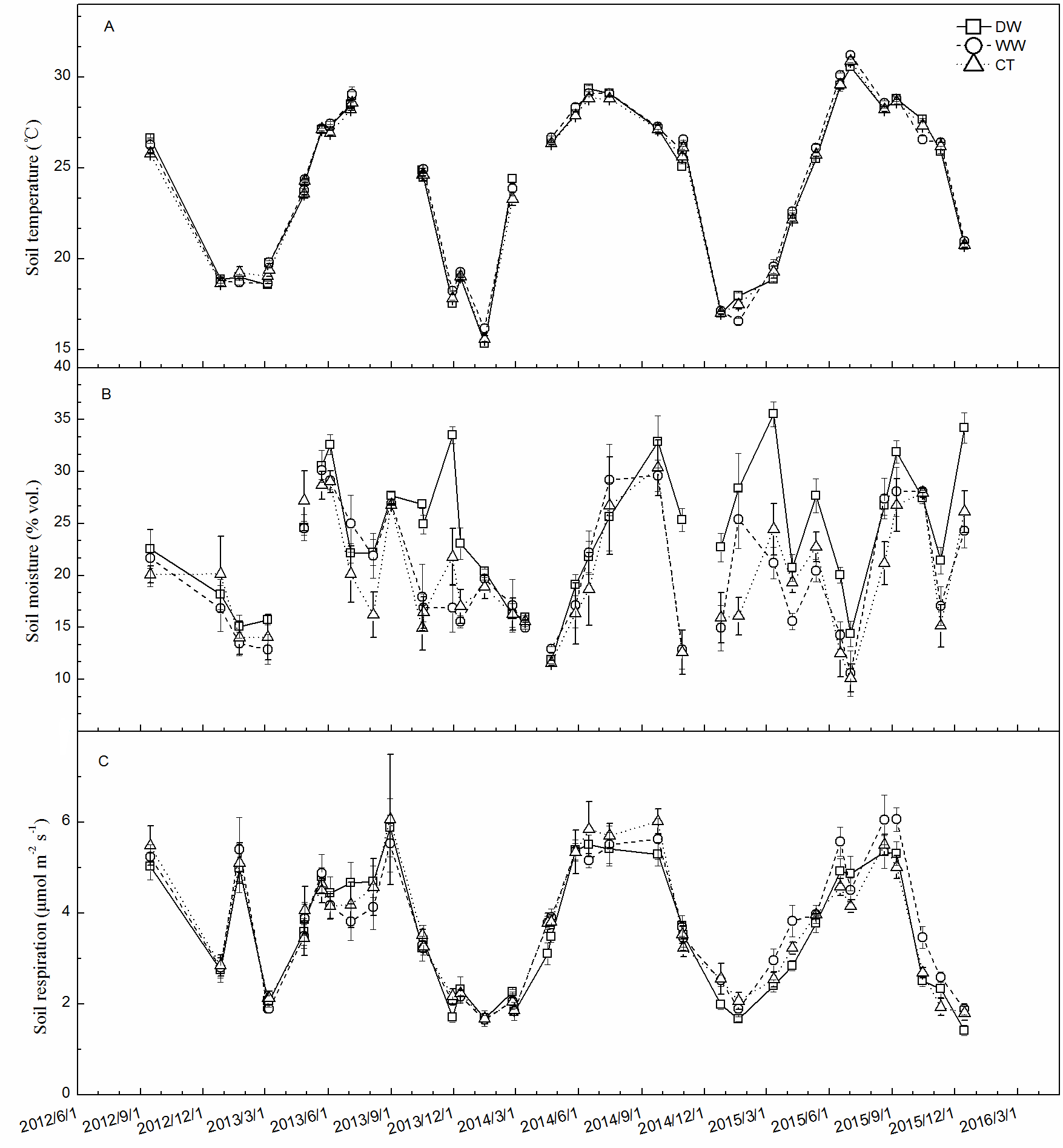


Figure S1 Seasonal dynamic of soil temperature (A), soil moisture (B) and soil respiration (C) from 2012 to 2015. Some measurements of soil temperature and soil moisture were missed because of instrument failure.


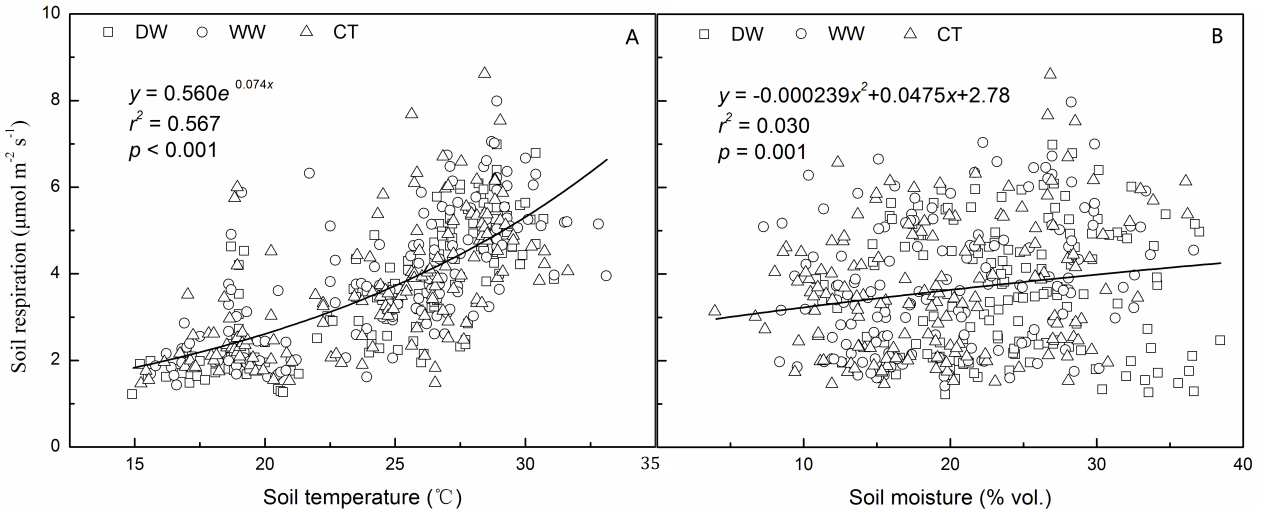


Figure S2 Exponential relationship between soil respiration and soil temperature (A) and soil moisture (B)

Table S1 Pearson correlation between soil moisture and other environmental variables in each period

|  | Soil moisture | | | |
| --- | --- | --- | --- | --- |
|  | January | March | August | November |
| LAI | *r* = 0.302 | *r* = 0.546 | *r* = -0.253 | *r* = -0.088 |
|  | *p* = 0.367 | *p* = 0.082 | *p* = 0.453 | *p* = 0.797 |
| UB | *r* = -0.306 | *r* = -0.118 | *r* = 0.439 | *r* = -0.003 |
|  | *p* = 0.333 | *p* = 0.714 | *p* = 0.154 | *p* = 0.993 |
| FRB | *r* = -0.135 | *r* = 0.207 | *r* = -0.502 | *r* = 0.126 |
|  | *p* = 0.676 | *p* = 0.518 | *p* = 0.096 | *p* = 0.696 |
| MBC | ***r* = 0.877** | ***r* = 0.615** | *r* = 0.366 | ***r* = 0.771** |
|  | ***p* < 0.001** | ***p* = 0.033** | *p* = 0.242 | ***p* = 0.003** |
| DOC | *r* = 0.341 | ***r* = 0.656** | *r* = 0.428 | ***r* = 0.714** |
|  | *p* = 0.278 | ***p* = 0.020** | *p* = 0.165 | ***p* = 0.009** |
| pH | *r* = -0.250 | *r* = -0.363 | *r* = 0.036 | ***r* = -0.616** |
|  | *p* = 0.434 | *p* = 0.247 | *p* = 0.912 | ***p* = 0.033** |
| SOM | *r* = -0.023 | |  |  |
|  | *p* = 0.944 | |  |  |
| FL | *r* = 0.068 | |  | *r* = 0.286 |
|  | *p* = 0.834 | |  | *p* = 0.368 |

LAI: leaf area index; UB: understory biomass (g m^-2^); FRB: fine root biomass (g m^-2^); MBC: microbial biomass carbon (mg kg^-1^ dry soil); DOC: dissolved organic carbon (mg kg^-1^ dry soil); FL: forest floor litter mass (g m^-2^); SOM: soil organic matter content (%). Significant correlation (*p* < 0.050) are highlighted in bold.
